# CARDIOVASCULAR AND MORTALITY RISK WITH COEXISTING HYPERTENSION, DYSLIPIDEMIA AND SYSTEMIC INFLAMMATION

**DOI:** 10.1093/geroni/igad104.3738

**Published:** 2023-12-21

**Authors:** Thomas Karadimas, Helen Meier

**Affiliations:** University of Michigan, Ann Arbor, Michigan, United States; University of Michigan, Ann Arbor, Michigan, United States

## Abstract

Hypertension and dyslipidemia are established risk factors for cardiovascular disease (CVD), but are often insufficient alone in predicting CVD. Inflammation also contributes to CVD, but research on the co-occurrence of inflammation, hypertension, and dyslipidemia and CVD risk is limited. Prospective data from the Health and Retirement Study, a representative cohort of US adults over 50 years of age (n = 1,527) were used. Hypertension, dyslipidemia, and elevated C-reactive protein (CRP) were used to create a CVD risk: low (0-1 factors), medium (2 factors), or high (all 3 factors). Weighted logistic regression models estimated the odds ratio (OR) of 1) prevalent and incident CVD for medium and high-risk groups versus the low-risk group and 2) 4-year mortality adjusting for covariates. Cross-sectionally, high-risk participants had significantly higher odds of CVD prevalence compared to participants with low-risk (adjusted OR = 1.68, 95% CI: [1.12 - 2.53]). Prospectively, medium and high-risk participants had higher odds of 4-year CVD incidence (medium: OR = 1.41, 95% CI: [0.71 - 2.79]; high OR = 2.05, 95% CI: [0.93 - 4.53]) compared to those with low-risk, but not statistically significant. Risk of 4-year mortality was higher in both medium (OR = 2.00 95% CI: [1.21 - 3.30]) and high-risk (OR = 2.11 95% CI: [1.15 - 3.86]) participants vs. low-risk. Co-occurrence of hypertension, dyslipidemia, and elevated CRP was associated with CVD prevalence and 4-year mortality in older US adults, emphasizing the importance of multifactor screening for CVD risk.

